# Double Trouble: Leishmaniasis and Cytomegalovirus Colitis in an Immunocompromised Host

**DOI:** 10.14309/crj.0000000000001687

**Published:** 2025-05-05

**Authors:** Saleh Alghaythi, Aymen Almuhaidb, Badr Al-Bawardy

**Affiliations:** 1 Department of Internal Medicine, Division of Gastroenterology and Hepatology, King Faisal Specialist Hospital, Riyadh, Saudi Arabia; 2 College of Medicine, Al-Faisal University, Riyadh, Saudi Arabia; 3 Department of Internal Medicine, Section of Digestive Diseases, Yale School of Medicine, New Haven, CT

**Keywords:** polyps, colonoscopy, gastroscopy, endoscopy, leishmaniasis, CMV colitis, renal transplant

## Abstract

Leishmaniasis is endemic in several areas of the world. It is a chronic protozoan disease transmitted through the bite of the Phlebotomus fly. Unusual localizations of visceral leishmaniasis are extremely rare and are described in immunocompromised patients. We present a case of a 73-year-old man, postliving nonrelated kidney transplant who complained of melena, fatigue, and weight loss. Gastroscopy showed a large ulcer at the lesser curvature of the stomach, and biopsies demonstrated severe ulceration with superimposed fungal organisms. Colonoscopy showed a polyp in the transverse colon, and histopathological examination revealed cytomegalovirus colitis with superimposed leishmaniasis.

## INTRODUCTION

Leishmaniasis is a chronic protozoan disease caused by a parasite of the species Leishmania and transmitted through the bite of the Phlebotomus fly. It is endemic in several areas of the world, including Saudi Arabia.^1^ Visceral leishmaniasis is a systemic disease characterized by hepatosplenomegaly, pyrexia, weight loss, elevated gammaglobulinemia, and pancytopenia.^2^ However, asymptomatic leishmanial infection has been reported previously.^2^ The incubation period is usually prolonged, and in state of decreased immunity, there is evidence of activation of latent infection few years after exposure to the parasite.^2^ Unusual localizations of visceral leishmaniasis include enteric involvement and are described in patients with adult immunodeficiency syndrome and in geriatric immunocompetent individuals.^3–5^ Establishing a diagnosis of enteric leishmaniasis requires visualization of amastigotes within the macrophages of intestinal lamina propria in histopathology.^4,6^

Infections have significant impact on mortality and morbidity in immunocompromised patients including renal allograft recipients.^7^ Immunosuppressive medications and immunomodulatory viruses including cytomegalovirus (CMV) predispose the recipients to many opportunistic infections.^8^ The coinfection of CMV with Leishmaniasis produces a diagnostic dilemma as clinical features overlap and might delay the diagnosis. We report a rare presentation of a renal transplant recipient who had coinfection of CMV and enteric visceral leishmaniasis presenting as gastric ulcerations and a colonic polyp.

## CASE REPORT

A 73-year-old man, resident of the central region of Saudi Arabia, was admitted for a complaint of continuous generalized fatigability, melena, and significant weight loss for 3 months. He had received a living nonrelated renal transplant in 2007 for end-stage renal disease secondary to obstructive uropathy-induced interstitial nephritis. Both recipient (R) and donor (D) were CMV IgG positive. Postoperative course was uncomplicated, and the patient was discharged on triple immunosuppressive agents (mycophenolate mofetil, tacrolimus, and prednisone taper). He had stable graft function since then on the same immunosuppressive agents with baseline creatinine of 92 mmol/L and blood urea nitrogen of 7 mmol/L. His medical history includes type 2 diabetes mellitus, hypertension, ischemic heart disease, and primary prostate hyperplasia.

He presented to the emergency department with a 3-month history of melena, generalized fatigability, and significant unintentional weight loss of about 16 kg. He denied fever/chills, abdominal pain, bleeding from any site, shortness of breath, or neurological symptoms. There were no changes in his medications in the past year. Social history was negative for alcohol intake, or high-risk behavior except for smoking. The family history was unremarkable for chronic gastrointestinal or infectious diseases.

On admission he was afebrile, not in pain or distress, alert and oriented, and not jaundiced or cyanosed. The abdomen was soft without tenderness, palpable masses, or organomegaly. Laboratory workup on admission revealed hemoglobin: 10.1 g/dL (matching baseline), mean corpuscular volume: 81 fL, white blood cell count: 7.3 cells/μL, platelets: 226 10^3^/µL; C-reactive protein: 214, procalcitonin: 0.16 ng/mL, alanine aminotransferase: 15 U/L; aspartate aminotransferase: 21.2 U/L, creatinine: 122 µmol/L, blood urea nitrogen: 12.8 mmol/L, CMV polymerase chain reaction: 75 IU/mL, albumin, and electrolytes were within normal limits.

**Figure 1.**
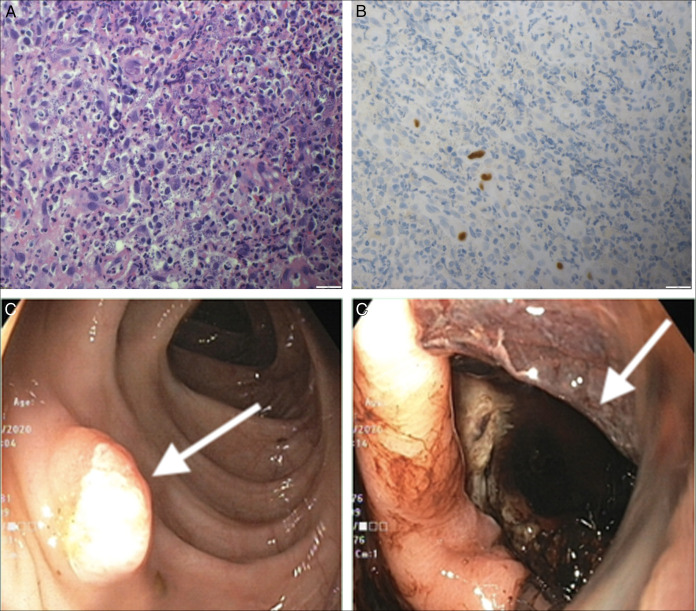
(A) Pathology slide (20×) from the transverse colonic polyp demonstrating the CMV colitis with superimposed leishmaniasis in hematoxylin plus eosin stain. (B) Pathology slide (20×) from the transverse colonic polyp showing the CMV colitis with superimposed leishmaniasis in immunohistochemistry stain. (C) Endoscopic image from the transverse colon showing a 1 cm sessile polyp. The arrow in C points at the sessile polyp in transverse colon. (D) Endoscopic image showing a gastric deep ulcer in greater curvature. The arrow in D points at gastric deep ulcer. CMV, cytomegalovirus.

Positron emission tomography was done investigating his constitutional symptoms and showed hyper-metabolic activity along gastric antrum with few subcentimetric mesenteric lymphadenopathy and 1 fluorodeoxyglucose-avid colonic lesion at the hepatic flexure. Gastroscopy was performed and showed a large deep ulcer extending along the gastric lesser curvature of about 4 cm in length and with irregular borders covered with a small clot (Figure 1). Colonoscopy showed a 1 cm sessile polyp in transverse colon (Figure 1). Gastric ulcer biopsy demonstrated severe ulceration and granulation tissue with superimposed fungal organisms; scattered granulomas were noted and stained negative for acid fast bacilli. It was negative for malignancy and intestinal metaplasia. Transverse colon polyp biopsy revealed CMV colitis with superimposed leishmaniasis (Figure 1). The patient was initiated afterward on amphotericin B, received a total of 11 doses (cumulative dose of 44 mg/kg), in conjunction with ganciclovir for 2 weeks, followed by valganciclovir for an additional 3 weeks. After 5 weeks of treatment, the patient demonstrated clinical and endoscopic improvement, with evidence of a healed gastric ulcer. Histopathology was unrevealing for any viral inclusions or leishmaniasis.

## DISCUSSION

Invasive enteric leishmaniasis can affect any part of the digestive system with the most common site being the duodenum.^9^ It can manifest with variety of clinical presentations and might as well be asymptomatic.^10^ It is crucial to reach diagnosis in patients with leishmaniasis as it has a fatality rate that can reach 95% in immunocompromised hosts if missed.^11^ Leishmaniasis is considered endemic in the southern areas of Saudi Arabia, and this might have contributed to delaying the diagnosis as the patient lived in the central region of the kingdom.

As noted in this case, the previously reported cases of intestinal localization of Leishmania were all in immunocompromised patients to acquired immunodeficiency syndrome, organ transplant, or elderly patients with comorbid conditions, which could lead to altered innate and T-cell-related immune response.^12,13^ Endoscopic examination may be normal in some cases and rarely presents as a colonic polyp.^14^ Biopsies needs to be obtained when diagnosis of enteric leishmaniasis is suspected. Rosenthalm et al observed that in 12 of 19 patients, the diagnosis of enteric leishmaniasis was confirmed only with gastrointestinal biopsies as endoscopic appearance was normal.^15^

Solid organ recipients are predisposed to many infections secondary to immunosuppressive medications. CMV infection has immunomodulating and immunosuppressant effect, which predisposes the recipient to other infections.^8^ Thus, it is possible that the CMV disease in this case triggered the parasitic infection activation. However, CMV may also be part of superinfections reported in patients with visceral leishmaniasis.^16^ The coinfection with CMV colitis and leishmaniasis is considered very rare and played a role in delaying the diagnosis, especially in the absence of splenomegaly in imaging as splenomegaly was observed in all immunocompetent patients with visceral leishmaniasis and about 86% of solid organ transplant recipients.^17^

Mycotic infections of the gastrointestinal tract although rare in immunocompetent individuals, it can be identified in up to 50% of gastric ulcers in immunosuppressed patients.^18^ In a recent review of 109 cases of gastrointestinal fungal infection, Eras et al reported that all but 2 patients had underlying malignancy.^19^ While esophageal fungal infections typically present as plaques, ulcerations are the most common extraesophageal manifestation of invasive fungal infections.^20^ The role of fungal organisms in gastric ulcers remains unclear, whether they represent mere colonization or contribute to pathogenesis is still debatable; nevertheless, in this case, the absence of fungal invasion on histological examination suggests that the presence of fungi is likely a secondary phenomenon. Furthermore, the potential involvement of fungal colonization in the development of gastrointestinal CMV disease or leishmaniasis remains uncertain. It is also unclear whether CMV plasma DNA reactivation increases the risk of invasive fungal infections, as existing literature presents conflicting findings.

In conclusion, CMV colitis superimposed with enteric leishmaniasis presenting as a colonic polyp is extremely rare.

However, it should always be considered in immunocompromised patients coming from endemic areas as delaying the diagnosis and management significantly increases mortality. This is particularly true if the patient was having constitutional or gastrointestinal symptoms (bleeding, diarrhea). Whenever enteric leishmaniasis is suspected, gastroenterologists should obtain biopsies and the pathologist should also be alerted to consider the potential presence of this parasite.

## DISCLOSURES

Author contributions: B. Al-bawardy: case selection, assisted with manuscript review and audit, Final approval of the version to be published; S. Alghaythi: case selection, literature review, full manuscript and abstract writing, publication efforts; A. Almuhaidb: case selection, case selection, assisted with manuscript review. B. Al-bawardy is the article guarantor.

Financial disclosure: Dr. Badr Al-Bawardy: Speaker fees: AbbVie, Takeda, Bristol-Myers Squibb, Janssen Pharmaceuticals. Advisory board: Bristol-Myers Squibb, Pfizer. All other authors have no financial disclosures.

Informed consent was obtained for this case report.

## References

[1] Soria López E Olalla Sierra J del Arco Jiménez A Colonic leishmaniasis in a patient with HIV: A case report. Rev Esp Enferm Dig. 2016;108(12):838-40. 26901148 10.17235/reed.2016.4038/2015

[2] Català A Roé E Dalmau J Anti-tumour necrosis factor-induced visceral and cutaneous leishmaniasis: Case report and review of the literature. Dermatology. 2015;230(3):204–7. 25633623 10.1159/000370238

[3] Fernández-Guerrero ML Robles P Rivas P Mójer F Muñíz G de Górgolas M. Visceral leishmaniasis in immunocompromised patients with and without AIDS: A comparison of clinical features and prognosis. Acta Trop. 2004;90(1):11–6. 14739017 10.1016/j.actatropica.2003.09.009

[4] Hernández JA Bosch MA Sauca G. Atypical clinical presentation of visceral leishmaniasis. Haematologica. 1999;84(8):750. 10457413

[5] Pasquau F Ena J Sanchez R ; Leishmania HIV Mediterreanean Co-operative Group. Leishmaniasis as an opportunistic infection in HIV-infected patients: Determinants of relapse and mortality in a collaborative study of 228 episodes in a Mediterreanean region. Eur J Clin Microbiol Infect Dis. 2005;24(6):411–8. 15928908 10.1007/s10096-005-1342-6

[6] Hernández M González-Lama Y González-Lama Y Ramos A Martínez-Ruiz R Calvo M. Visceral leishmaniasis as an unusual infectious complication in a patient with Crohn’s disease treated with infliximab. Gastroenterol Hepatol. 2015;38:411–2. 25443540 10.1016/j.gastrohep.2014.08.003

[7] Peterson PK Anderson RC. Infection in renal transplant recipients. Current approaches to diagnosis, therapy, and prevention. Am J Med. 1986;81(1A):2–10. 10.1016/0002-9343(86)90509-7 3090876

[8] Berenguer J Gomez-campdera F Padilla B, et al. Visceral leishmaniasis (Kala-azar) in solid organ transplants: Case report and review. Transplantation. 1998;65:1401–4. 9625028 10.1097/00007890-199805270-00022

[9] Alvar J Cañavate C Gutiérrez-Solar B Leishmania and human immunodeficiency virus coinfection: The first 10 years. Clin Microbiol Rev. 1997;10(2):298–319. 9105756 10.1128/cmr.10.2.298 PMC172921

[10] Jawhar NM. Visceral leishmaniasis with an unusual presentation in an HIV positive patient. Sultan Qaboos Univ Med J. 2011;11(2):269–72. 21969901 PMC3121034

[11] Bhatia IPS Tripathi S Singh A Hasvi J Rajan A Tukaram DV. Co-infection of cytomegalovirus and Leishmania without splenomegaly resulting in immunosuppression in an HIV-negative patient. Eur J Case Rep Intern Med. 2024;11:004923. 39525445 10.12890/2024_004923 PMC11542958

[12] Eaton S Burns E Kusser K Randall T Haynes L. Age-related defects in CD4 T cell cognate helper function lead to reductions in humoral responses. J Exp Med. 2004;200:1613–22. 15611289 10.1084/jem.20041395 PMC2211991

[13] d'Ettorre G Ceccarelli G Carnevalini M Central role of interleukin-15 in human immunodeficiency virus (HIV)-infected patients with visceral leishmaniasis. Acta Trop. 2006;99(1):83–7. 16962979 10.1016/j.actatropica.2006.08.002

[14] Zaffiri L d'Ettorre G Massetti AP Mascellino MT Mastroianni CM Vullo V. Atypical localization of Leishmaniasis in an intestinal polyp. Infection. 2008;36(2):187–8. 18327682 10.1007/s15010-007-7038-3

[15] Rosenthal E Marty P Del Giudice P HIV and leishmania coinfection: A review of 91 cases with focus on atypical locations of leishmania. Clin Infect Dis. 2000;31(4):1093–5. 11049794 10.1086/318135

[16] Ersoy A Güllülü M Usta M A renal transplant recipient with pulmonary tuberculosis and visceral leishmaniasis: Review of superimposed infections and therapy approaches. Clin Nephrol. 2003;60(4):289–94. 14579946 10.5414/cnp60289

[17] Bryceson AD. Leishmaniasis. In: Cook GC Zumla AI, eds. Manson’s Tropical Diseases. 20th ed. London: WB Saunders; 1996:1213–45.

[18] Loffeld RJ Loffeld BC Arends JW Flendrig JA van Spreeuwel JP. Fungal colonization of gastric ulcers. Am J Gastroenterol. 1988;83(7):730–3. 3381804

[19] Eras P Goldstein MJ Sherlock P. Candida infection of the gastrointestinal tract. Medicine (Baltimore). 1972;51(5):367–79. 4560040 10.1097/00005792-197209000-00002

[20] Rai P Chakraborty SB. Giant fungal gastric ulcer in an immunocompetent individual. Saudi J Gastroenterol. 2012;18(4):282–4. 22824773 10.4103/1319-3767.98438 PMC3409891

